# Crystal Structures of Penicillin-Binding Protein 3 from *Pseudomonas aeruginosa*: Comparison of Native and Antibiotic-Bound Forms

**DOI:** 10.1016/j.jmb.2010.10.024

**Published:** 2011-01-07

**Authors:** Sarah Sainsbury, Louise Bird, Vincenzo Rao, Sharon M. Shepherd, David I. Stuart, William N. Hunter, Raymond J. Owens, Jingshan Ren

**Affiliations:** 1 Division of Structural Biology, Henry Wellcome Building for Genomic Medicine, University of Oxford, Roosevelt Drive, Oxford OX3 7BN, UK; 2 Oxford Protein Production Facility UK, The Research Complex at Harwell, Rutherford Appleton Laboratory Harwell Science and Innovation Campus, Oxfordshire OX11 0FA, UK; 3 Biological Chemistry and Drug Discovery, College of Life Sciences, The Wellcome Trust Building, University of Dundee, Dow Street, Dundee DD1 5EH, UK

**Keywords:** PBP, penicillin-binding protein, HMM, high molecular mass, LMM, low molecular mass, PDB, Protein Data Bank, ESRF, European Synchrotron Radiation Facility, anti-bacterial, *Pseudomonas aeruginosa*, carbenicillin, ceftazidime, enzyme structure

## Abstract

We report the first crystal structures of a penicillin-binding protein (PBP), PBP3, from *Pseudomonas aeruginosa* in native form and covalently linked to two important β-lactam antibiotics, carbenicillin and ceftazidime. Overall, the structures of apo and acyl complexes are very similar; however, variations in the orientation of the amino-terminal membrane-proximal domain relative to that of the carboxy-terminal transpeptidase domain indicate interdomain flexibility. Binding of either carbenicillin or ceftazidime to purified PBP3 increases the thermostability of the enzyme significantly and is associated with local conformational changes, which lead to a narrowing of the substrate-binding cleft. The orientations of the two β-lactams in the active site and the key interactions formed between the ligands and PBP3 are similar despite differences in the two drugs, indicating a degree of flexibility in the binding site. The conserved binding mode of β-lactam-based inhibitors appears to extend to other PBPs, as suggested by a comparison of the PBP3/ceftazidime complex and the *Escherichia coli* PBP1b/ceftoxamine complex. Since *P. aeruginosa* is an important human pathogen, the structural data reveal the mode of action of the frontline antibiotic ceftazidime at the molecular level. Improved drugs to combat infections by *P. aeruginosa* and related Gram-negative bacteria are sought and our study provides templates to assist that process and allows us to discuss new ways of inhibiting PBPs.

## Graphical abstract

**Figure:**
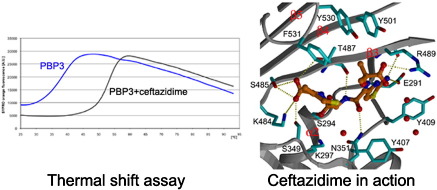

## Introduction

The bacterial cell wall consists of *N*-acetylglucosamine-*N*-acetyl muramic polymers cross-linked *via* the penultimate d-alanine residues of muramyl pentapeptides to form a peptidoglycan network that is essential for normal cell survival. Penicillin-binding proteins (PBPs) are membrane-bound enzymes involved in the final stages of bacterial cell wall synthesis on the periplasmic side of the membrane. They have been classified into a high-molecular-mass (HMM) group, members of which are essential for cell viability, and a low-molecular-mass (LMM) group, members of which appear dispensable for normal cell growth.^1^ HMM PBPs are further divided into class A enzymes, which catalyze both the polymerization of a peptidoglycan from disaccharide peptides (glycosyltransferase) and the cross-linking of muramyl peptides (transpeptidase), and class B enzymes, which only possess transpeptidase activity. HMM PBPs consist of two domains: an N-terminal membrane-proximal domain and a C-terminal domain harboring transpeptidase activity. In the case of class A PBPs, the N-terminal domain contains glycosyltransferase activity; the function of the equivalent domain in class B PBPs is not known. The smaller LMM PBPs are d,d-carboxypeptidases that remove the terminal d-alanine from the muramyl peptide, thus controlling the level of peptidoglycan cross-linking.^1^ As the name indicates, PBPs are also the primary targets of β-lactam antibiotics, which act as suicide substrates by mimicking the d-alanyl-d-alanine stem peptide of peptidoglycan precursors. β-Lactams block the activity of transpeptidases and carboxypeptidases by acylating the active site serine residue.^2^

Crystal structures have been reported for HMM PBPs from the Gram-negative *Escherichia coli* (PBP1b)^3^ and *Neisseria gonorrhoeae* (PBP2),^4^ and the Gram-positive *Streptococcus pneumoniae* (PBP1b,^5^ PBP2x,^6–10^ and PBP2b^11^) and *Staphylococcus aureus* (PBP2a^12^), including both apo-enzymes and complexes with β-lactams. All of these structures show similar active sites, featuring three conserved catalytic motifs in the transpeptidase domain. The β-lactams are covalently bound to the active site serine *via* the carbonyl of the opened cyclic amide. Resistance to β-lactams can be rationalized to some extent by examining the structures of PBPs from resistant bacteria, which have mutations in residues close to the catalytic motifs.^2,7,11^

Our interest is primarily *Pseudomonas aeruginosa*, an opportunistic pathogen that is responsible for severe life-threatening infections, especially in patients suffering from chronic respiratory diseases such as cystic fibrosis.^13^ The genome of *P. aeruginosa* strain PAO1^14^ encodes four HMM PBPs, including one class A enzyme (PBP1a) and three class B enzymes (PBP2, PBP3, and PBP3a), which are orthologues of the corresponding enzymes in *E. coli*. PBP3 shares 42% sequence identity with the corresponding protein in *E. coli* and has been identified as the primary target of a number of β-lactams used to treat pseudomonal infections, including the cephalosporin analogues cefsulodin^15^ and ceftazidime,^16^ piperacillin,^17^ and the parenteral carbapenem, doripenem.^18^

Compared to many other Gram-negative bacteria, *P. aeruginosa* is highly resistant to antibiotics and, as with many bacterial pathogens, resistance increases with repeated use and misuse of antibiotics. As is typical of Gram-negative bacteria, antibiotic resistance in *P. aeruginosa* is largely due to a combination of β-lactamase production and the action of numerous efflux pumps.^19^ However, treatment with high doses of piperacillin has been shown to be associated with reduced binding to PBPs in clinical isolates of *P. aeruginosa*.^17^ In laboratory strains lacking β-lactamases, the overproduction of PBP3 in *P. aeruginosa* promotes reduced sensitivity to cefsulodin;^20^ in a separate study, increased resistance to cefsulodin was observed to be associated with reduced binding to endogenous PBP3.^15^

PBP3 is a therapeutic target in *P. aeruginosa*, and we have determined structures of this HMM PBP in its native form and in complex with two relevant drugs, carbenicillin and ceftazidime (Supplementary Material, Fig. S1). Carbenicillin is a broad-spectrum semisynthetic penicillin derivative and was one of the first derivatives to display significant activity against *Pseudomonas*.^21^ The aminothiazolyl-carrying ceftazidime^16^ is similar to cefotaxime, the first third-generation cephalosporin to be marketed, but differs with a 2-carboxy-2-oxypropaneimino group replacing a methoxy amino group. This difference increases efficacy against *P. aeruginosa*, making ceftazidime one of only a handful of drugs currently used to treat *Pseudomonas* infections. Ceftazidime also displays potent activity against another of the so-called superbugs, *Burkholderia pseudomallei*, which was previously known as *Pseudomonas pseudomallei* and is the causal agent of melioidosis, a serious infection in humans found mainly in Asia. Indeed, ceftazidime is the frontline drug for the treatment of this disease.

We describe the crystal structures of apo-PBP3 and the acyl complexes with carbenicillin and ceftazidime, as well as the similarities and differences between the three structures, and discuss structural features that might be exploited to derive novel inhibitors of therapeutic value.

## Results and Discussion

### Production of PBP3 and binding to β-lactams

**Fig. 1:**
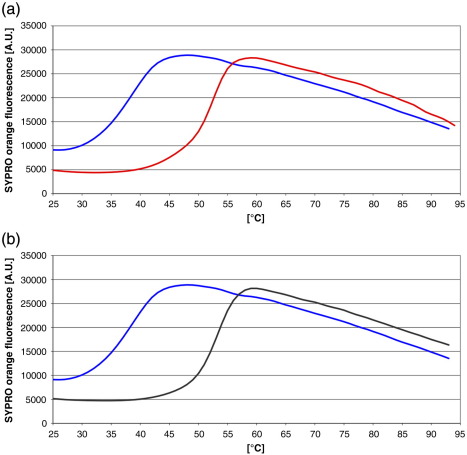
Thermal shift assay. (a) PBP3 screened with 500 μM carbenicillin, giving a Δ*T*_m_ of + 13.2 °C. The blue curve is the native protein, and the red curve is the complex. (b) PBP3 screened with 500 μM ceftazidime, giving a Δ*T*_m_ of + 14.5 °C. The blue curve is the native protein, and the black curve is the complex.

A soluble version of recombinant *P. aeruginosa* PBP3 was produced by truncating the sequence at the N-terminus by 34 residues to remove the predicted transmembrane helix. The molecular mass of the purified protein determined by size-exclusion chromatography was approximately 54 kDa compared to a calculated value of 61 kDa, indicating that it is most likely a monomer in solution. Binding of carbenicillin and ceftazidime to the recombinant protein was confirmed using a thermal shift assay. The assay used a real-time PCR machine to detect changes in fluorescence during the thermal unfolding of the protein in the presence of SYPRO Orange. Significant temperature stabilization of the protein was observed for both compounds, with shifts in the midpoint transition temperature (Δ*T*_m_) of 13.2 ± 0.2 °C and 14.5 ± 0.1 °C following incubation with either 0.5 mM carbenicillin or 0.5 mM ceftazidime, respectively (Fig. 1).

### Overall structure

Three isomorphous structures of *P. aeruginosa* PBP3 have been determined: apo-PBP3 at 2.0 Å resolution, PBP3/carbenicillin complex at 2.4 Å resolution, and PBP3/ceftazidime complex at 2.6 Å resolution. The PBP3/carbenicillin complex was derived from a sample obtained by cocrystallization, and the ceftazidime complex was derived by soaking a native crystal in mother liquor containing the antibiotic. The initial structure was determined using the coordinates of PBP2 from *N. gonorrhoeae*^4^ as molecular replacement model [Protein Data Bank (PDB) code 3equ]. Crystallographic statistics (Table 1) indicate that acceptable models have resulted from the analyses. In all three structures, residues 35–49, 492–497, and 560–577 are disordered and do not have sufficient electron density to allow reliable model building.

**Fig. 2:**
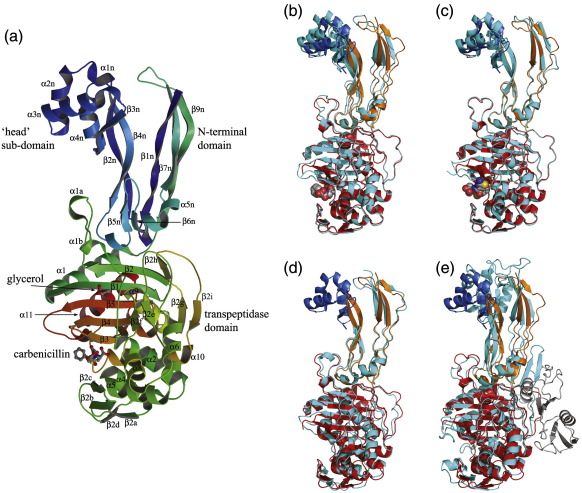
The overall structure of PBP3. (a) A ribbon diagram of the PBP3/carbenicillin complex showing the overall fold, antibiotic binding site, and bound glycerol. The diagram is rainbow-colored from blue at the N-terminus to red at the C-terminus, and the secondary structure elements are labelled in accordance with previous studies.^15,16^ (b–e) A comparison of the overall structure of native PBP3 (blue, orange, and red) with those of the PBP3/carbenicillin complex (b), PBP3/ceftazidime complex (c), PBP2 of *N. gonorrhoeae* (d), and PBP2x from *S. pneumoniae* (e). This figure was produced with PyMOL (www.pymol.org). The rest of the figures were drawn using BOBSCRIPT.^22^

PBP3 is a two-domain protein comprising a C-terminal transpeptidase linked to an extended N-terminal domain. Overall, PBP3 resembles other class B PBPs, although it lacks the additional C-terminal domain unique to the PBP2x structure (Fig. 2). In common with these other class B PBPs, there is an α-helical subdomain or “head” domain^11^ (residues 80–149) towards the N-terminus, which was not resolved in the PBP2 structure (Fig. 2 and Table 2). The function of the N-terminal domain of class B PBPs is not known but possibly serves to position the transpeptidase domain away from the inner membrane as part of a multienzyme complex involved in cell wall biosynthesis.^24^

Despite a relatively low sequence identity, the fold of the transpeptidase domain is highly conserved among PBPs. For the class B enzymes, the RMSD for the overlap of approximately 80% of the C^α^ atoms is 1.5–2.0 Å, depending on the structure (Table 2). The structural superimpositions also revealed that the orientation of the transpeptidase domains varied with respect to the N-terminal domains in the different structures. For example, in the overlay of the PBP3 and PBP2b transpeptidase domains, the N-terminal domains of the PBPs were rotated as rigid bodies by about 24° with respect to each other (Table 2), indicating considerable rotational freedom between the N-terminal domain and the C-terminal domain.

### Active site structure

**Fig. 3:**
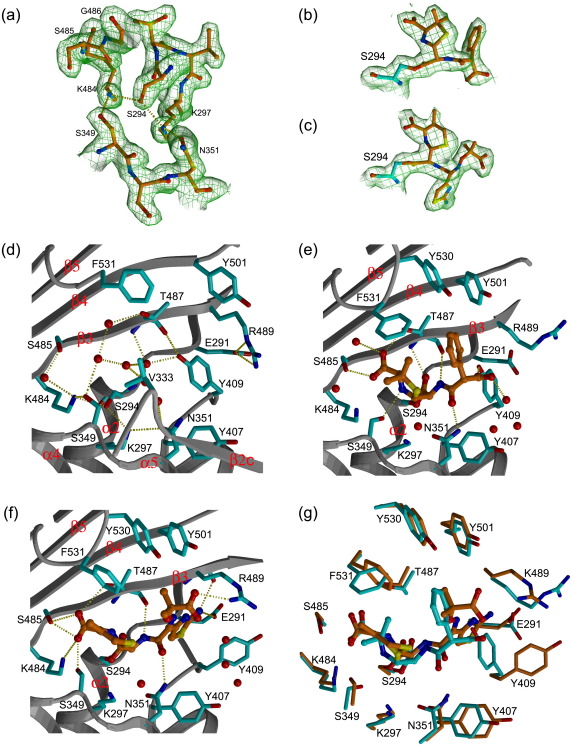
The active site of PBP3. (a–c) 2*F*_o_ − *F*_c_ maps calculated after the final round of refinement and contoured at 1σ showing the electron density for the three active site structural motifs in apo-PBP3, bound carbenicillin, and ceftazidime in the two complexes, respectively. In (b) and (c), the carbon atoms of the acylated S294 are shown in cyan. (d–f) The active sites of apo-PBP3, carbenicillin, and ceftazidime complexes. The protein main chains are shown as gray ribbons, side chains are drawn as cyan sticks, and the inhibitor is shown in orange ball-and-sticks. The red spheres represent water molecules, and the yellow broken lines represent potential hydrogen bonds. Residue V333 that makes contacts with the bound inhibitors is not shown in (e) and (f) for clarity. (g) Comparison of the active sites of the PBP3/carbenicillin complex (cyan) and the PBP3/ceftazidime complex (orange).

The active site of PBP3 is located in a long cleft running parallel with the β3 strand across the lower part of the transpeptidase domain. The three conserved structural motifs that are common to d,d-peptidases and β-lactamases are found here and have well-defined electron density (Fig. 3a). The SXXK motif (S294-T295-V296-K297) is located at the N-terminal end of α2 and includes the nucleophilic S294, together with the α8–β2f loop (residues 407–409) that forms the base of the cleft. The SXN motif (S349-S350-N351) occurs in the α4–α5 turn, and residues 328–334 (β2c and β2c–β2d loop) form one side of the elongated cleft; the third motif KS/TG (K484-S485-G486) is positioned on strand β3 and, together with residues 530–533 in the β5–α11 loop, forms the opposite side of the cleft. Unlike in PBP2a,^12^ which is relatively resistant to β-lactam inhibition, the catalytic S294 is positioned favorably for acylation in an open substrate-binding pocket. The three conserved structural motifs interact with each other *via* a network of hydrogen bonds: the hydroxyl group of S294 forms hydrogen bonds with the side chains of both K297 and K484 (2.7 Å and 2.8 Å), which are hydrogen bonded to S349 and N351, respectively (Fig. 3d). The position of K297 makes it one of the possible proton receptors from the catalytic S294 during the acylation reaction, in addition to the possible contribution of the β-lactam carboxylate. There are eight water molecules in the active site cleft of apo-PBP3, which form an extensive hydrogen-bonding network with the protein. The two loops connecting β3 and β4 (K490-A500) and β5 and α11 (S526-F531) are disordered in the apo-enzyme.

### Comparison of apo-enzyme and acyl-enzyme complexes

**Fig. 4:**
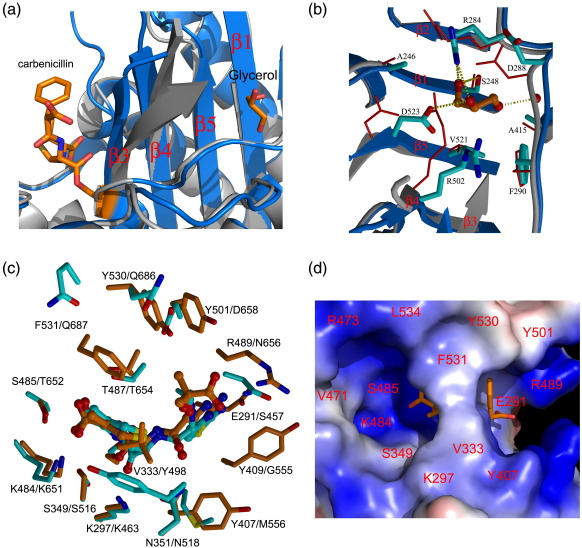
Glycerol binding, comparison with PBP1b, and electrostatic properties of the active site. (a) A diagram showing that the glycerol binding site is separated from the inhibitor binding site by β3–β4 strands and large conformational changes of the two strands due to inhibitor binding. The main-chain backbones of apo-PBP3 and the PBP3/carbenicillin complex are shown as gray and blue ribbons, respectively. (b) Comparison of the glycerol-binding site in the PBP3/carbenicillin complex with that in the apo-enzyme. The main chains and side chains are shown as ribbons and sticks, respectively (in gray and red for apo-PBP3; in blue and cyan for the carbenicillin complex), and the glycerol molecule is drawn as orange sticks. (c) Comparison of the active sites of the PBP3/ceftazidime complex (orange) and the *S. pneumoniae* PBP1b/cefotaxime complex (cyan). (d) Electrostatic surface showing the inhibitor-binding pocket of the PBP3/carbenicillin complex. (a) and (c) were produced with PyMOL (www.pymol.org).

In comparing the apo-PBP3 and acyl-PBP3 complexes, it is apparent that the N-terminal domains show greater structural flexibility than the C-terminal transpeptidase domains. Thus, the superposition of the N-terminal β subdomains (residues 50–79 and 150–221) of the PBP3/carbenicillin and PBP3/ceftazidime complexes with the apo-enzyme resulted in RMSDs of 1.3 Å and 1.1 Å for 166 and 169 equivalent C^α^ atoms, and 7° and 5° differences in orientation for the head subdomains, respectively. By contrast, the C-terminal transpeptidase domains of both acyl-PBP3 enzymes superimpose on the apo-enzyme with RMSDs of 0.8 Å and 0.6 Å for the carbenicillin complex and the ceftazidime complex for 319 of 324 overlapped C^α^ positions. This shows that acylation does not significantly alter the overall conformation of the transpeptidase domain. However, significant local changes are observed in the substrate-binding pocket with movement of β3 relative to α2, reinstating the strict anti-parallel arrangement of the three β-strands (β3, β4, and β5). Strands β3 and β4 of PBP3 bend towards the inhibitor around a hinge region located at residue G486 on β3 and at residue L504 on β4, resulting in an approximately 4-Å movement for the C^α^ atoms of K490 and A500, the last and the first ordered residues of the β3 and β4 strands in the apo structure, respectively (Fig. 4a). A similar movement of the equivalent strands has been observed following the binding of β-lactams to other HMM^5^ and LMM PBPs.^25^ Ligand binding is associated with an increased order of the β3–β4 and β5–α11 loops, as shown by better electron density for these residues in the two complexes. Both carbenicillin and ceftazidime display well-defined electron density in the acylated enzyme complexes (Fig. 3b and c). The β-lactams are covalently bound to the active site S294 and adopt similar orientations that are maintained by hydrogen-bonding interactions between S485 and T487 and a carboxylate group on both compounds (Fig. 3d and e). The alignment of the drugs with T487 resembles a short segment of anti-parallel β-sheet. Note that ceftazidime is susceptible to hydrolysis,^26^ and since the R_2_ piperidine moiety of ceftazidime is lost on β-lactam ring opening, both compounds have similar chemical groups at this position [i.e., one methyl (ceftazidime) or two methyls (carbenicillin)], and these are positioned near V333 and F531 (Fig. 3g). The principal difference between the inhibitors involves the nature and the size of the R_1_ substituent (Fig. 3b and c; Supplementary Material, Fig. S1). In both complexes, the R_1_ amide linkage forms hydrogen bonds with the main-chain carbonyl of T487 and with the side chain of N351, and a cluster of aromatic residues (Y501, Y530, and F531) forms hydrophobic contacts with the R_1_ groups (Fig. 3e and f). In the carbenicillin complex, the R_1_ carboxylate forms hydrogen bonds with the side-chain hydroxyl of Y409 and a water molecule, which in turn interacts with the main-chain amide of R489. In order to accommodate the much larger R_1_ substituent on ceftazidime, the side-chain of Y409 is rotated away from the position observed in the apo-structure. This movement creates a subpocket where van der Waals interactions involving E291, G293, and A488 help to position the R_1_ aminothiazole ring and allows ring-stacking interactions with Y409. The position of the aminothiazole ring is also stabilized through bifurcated hydrogen bonds to the main-chain carbonyl of R489 and to the side-chain of E291, which is the only negatively charged residue in the cleft (Fig. 3g). The main-chain amide of R489 donates a hydrogen bond to the thiazole nitrogen. In the ceftazidime complex, the side-chain of R489 is rotated towards the ligand, allowing the side chain to donate a hydrogen bond to the R_1_ dimethyl/carboxylate group (Fig. 3f and g).

### Conservation of active-site residues in *B. pseudomallei* PBP3

As mentioned earlier, ceftazidime is used to treat infections with *B. pseudomallei*. The amino acid sequences of PBP3 from *P. aeruginosa* and *B. pseudomallei* were aligned (accession numbers Q51504 and Q63QJ1, respectively; data not shown). The enzymes share an approximately 40% identity. Of 12 residues (in and around the active site) that are critical for *P. aeruginosa* PBP3 reactivity and interaction with ceftazidime (K297, V333, S349, N351, Y409, K484, S485, T487, R489, Y501, Y530, and F531), 9 are strictly conserved. The differences are that V333 changes to aspartate, R489 changes to tyrosine, and Y530 changes to histidine. These are sterically conservative changes (e.g., the aliphatic component of the aspartate side-chain matches closely to a part of valine) and suggest that our model of the PBP3/ceftazidime complex reveals the structural basis for the action of ceftazidime in the treatment of melioidosis. Furthermore, this model provides a template for informing inhibitor development.

### Active site flexibility and variations

A common feature observed in the crystal structures of PBPs is the flexibility of the active sites, especially the loop linking β3–β4. This may be an intrinsic property of these enzymes that is required for substrate binding and release during the catalytic process. Active site flexibility has also been suggested to be a key mechanism for acquisition of drug resistance.^11^ Comparison of the structure of antibiotic-sensitive wild-type PBPs with those from resistant strains has shown that mutations occur in residues close to but not actually in the active site.^4,11^ The conformational change in β3 and β4 strands introduced by inhibitor binding opens up a small pocket on the other side of the β-sheet that is occupied by water molecules in apo-PBP3 (Fig. 4a). The crystal of the PBP3/carbenicillin complex had been soaked with cryoprotectant solution containing 20% glycerol prior to freezing (no cryoprotectant was used for apo and ceftazidime-soaked crystals). As a result, the pocket is occupied by a well-ordered glycerol molecule, which makes hydrogen-bonding interactions with S248, R284, D523, and a trapped water molecule, and hydrophobic contacts with residues G247, S248, F290, and V521 (Fig. 4b). This pocket could be targeted for the design of a new type of inhibitor that restricts active site “breathing.” The influence of such a ligand could be to increase the free energy of PBP3 ligand binding by restricting the conformational flexibility of the enzyme.^27^ A potential benefit could be use in conjunction with β-lactams to combat drug resistance.

Apart from the three conserved active site motifs, residues lining the substrate-binding cleft of PBPs are poorly conserved. Amino acid insertions and deletions often occur among PBPs and result in variations in the shape and size of the active site clefts. For example, *S. pneumoniae* PBP2x has an extended β3–β4 loop and a shorter linker between β5 and α11, resulting in an active-site cleft that is wider at one end and narrower at the other end compared to PBP3. The structure of the complex of *S. pneumoniae* PBP1b with cefotaxime, a close analogue of ceftazidime, has been reported,^11^ enabling a direct comparison of binding modes with the PBP3 complex. Superimposition of the complexes reveals that the two inhibitors dock into their respective targets in a remarkably similar way. The orientations of the residues that form hydrogen bonds with the common β-lactam core of the inhibitors, namely S485 (PBP3)/T652 (PBP1b), T487 (PBP3)/T654 (PBP1b), and N351 (PBP3)/N518 (PBP1b), are closely matched (Fig. 4c). N656 (PBP1b) and R489 (PBP3), which interact with the R_1_ groups of cefotaxime and ceftazidime, respectively, are also closely aligned, with their relative positions determined by the size of the R_1_ substituents. These observations point to a common binding mode for cephalosporin analogues to the HMM PBPs. Residues E291, Y409, and R489 in PBP3, which make key interactions with the R_1_ groups of ceftazidime, are replaced in PBP1b by S457, G558, and N656, respectively, likely reducing the affinity of PBP1b for the inhibitor given the different nature of these residues. More generally, sequence variation at these three positions could be a major factor contributing to the different specificities and affinities of cephalosporin analogues across PBPs.

Finally, it is striking that in all published structures of PBP/inhibitor complexes, the inhibitors occupy the central portion of the active site pocket, leaving a large volume of the cleft unoccupied (Fig. 4c and d). It may be possible to design inhibitors that utilize these “free” volumes to increase protein–inhibitor interactions and/or to prevent β-lactamase hydrolysis. Such modifications offer the potential of enhancing binding affinity for the target and of overcoming drug resistance caused by β-lactamase production.

## Experimental Procedures

### Protein production

A truncated version of the *ftsI* gene, omitting the bases encoding the first 34 amino acids (residues D35-G579), was amplified by PCR using *P. aeruginosa* PAO1 strain genomic DNA (ATCC strain 15692; LGC Standards Office, UK). The template and primers were designed for ligation-independent cloning *via* In-fusion™ technology (Clontech). PCR with KOD HiFi™ polymerase (Merck) used the following forward and reverse primers: 5′-aagttctgtttcagggcccgGACCTGCACGTGATCGACC-3′ and 5′-atggtctagaaagctttaGCCACGCCCTCCTTTTGC-3′, respectively. The PCR product was purified using paramagnetic microparticles^28^ (Agencourt AmPure™ System; Beckman Coulter) and inserted into the vector pOPINF using the protocol of Berrow *et al.*^29^ Recombinant clones were identified by PCR with the gene-specific forward primer and a T7 reverse primer, and verified by DNA sequencing. Native PBP3 was produced in the *E. coli* strain Rosetta *p*LysS (DE3) using autoinduction.^30^ Cells were grown in 2-L cultures of Overnight™ Express Instant TB media (Merck) at 37 °C for 4 h, and the temperature was then lowered to 25 °C. After incubation for a further 20 h, the cells were harvested by centrifugation for 15 min at 6000*g* and lysed using a Basic-Z Cell Disruptor (Constant Systems) at 30,000 psi in the presence of 50 mM Tris–HCl (pH 7.5), 500 mM NaCl, 0.2% Tween-20, 10 μg/ml DNase, and an ethylenediaminetetraacetic-acid-free Protease Inhibitor Cocktail Tablet (Roche). The lysate was centrifuged at 16,000*g* for 30 min to remove cell debris before the soluble fraction was loaded onto a 1-ml HisTrap FF column (GE Healthcare). The column was washed with 50 mM Tris–HCl (pH 7.5), 500 mM NaCl, and 20 mM imidazole before the elution of the protein in 50 mM Tris–HCl (pH 7.5), 500 mM NaCl, and 500 mM imidazole. The protein was then injected onto a 16/60 HiLoad™ Superdex 75 column (GE Healthcare) and eluted in 20 mM Tris–HCl (pH 7.5) and 200 mM NaCl. Protein-containing fractions were analyzed by SDS-PAGE (NuPage; Invitrogen) and combined, and the NaCl concentration was adjusted to 400 mM to improve protein solubility. Protein (4 mg/ml) was flash frozen and stored at − 80 °C.

### Thermal shift assay

A thermal shift assay,^31,32^ using the fluorescent dye SYPRO Orange (Invitrogen), was employed to demonstrate that the recombinant PBP3 binds the β-lactams and to determine their effect on protein stability. PBP3 was screened with carbenicillin and ceftazidime. Solutions of PBP3 at 4 μM, 1000× SYPRO Orange, and compounds at 500 μM concentration were made, and 40 μl was added to a 96-well thin-wall PCR plate (Thermo Scientific). The plates were sealed with adhesive PCR seal (4titude) and heated in an Mx3005p qPCR machine (Stratagene) from 25 °C to 95 °C at a rate of 1 °C/min. Fluorescence changes were monitored with excitation and emission wavelengths of 492 nm and 610 nm, respectively. Reference wells (i.e., solutions consisting only of PBP3 and the dye) were used to compare the melting temperature (*T*_m_) values. In order to rule out any effects caused by the presence of buffer, we used wells with buffer and 1000× SYPRO Orange as controls. Experiments were carried out in triplicate, and nonlinear regression analysis was determined in the curve-fitting program Prism (GraphPad Software). *T*_m_ values were calculated for each well and compared to the reference *T*_m_ values to obtain Δ*T*_m_ for each compound. A Microsoft Excel script† was used for convenient observations and comparison of melting curves and thermal shifts.

### Crystallization, data collection, and structure determination

Protein crystallization was carried out using standard Oxford Protein Production Facility protocols.^33^ Native crystals were grown by sitting-drop vapor diffusion at room temperature in 25% (wt/vol) polyethylene glycol 3350 and 0.1 M 2-[bis(2-hydroxyethyl)amino]-2-(hydroxymethyl)propane-1,3-diol propane (pH 7.8) containing 1% (wt/vol) protamine sulfate (Silver Bullet Screen; Hampton Research). Acyl complexes were obtained by either cocrystallization or soaking experiments. Crystals of the carbenicillin complex were obtained in 20% (wt/vol) polyethylene glycol 3350 and 0.2 M disodium tartrate containing 0.5 mM carbenicillin. The PBP3/ceftazidime complex was obtained by soaking native crystals in the crystallization solution containing 10 mM ceftazidime for 30 min at room temperature. All crystals were flash frozen in liquid nitrogen and then kept at − 173 °C under a stream of nitrogen gas during data collection. No cryoprotectant was used apart from the carbenicillin complex, where a crystal was soaked in solution containing 20% glycerol (vol/vol) and 80% of the reservoir solution for a few seconds before freezing. X-ray data were collected for the carbenicillin complex on beamline ID23.2 of the European Synchrotron Radiation Facility (ESRF; Grenoble, France), and data sets from the native and ceftazidime-soaked crystals were collected on beamlines I03 and I02 of the Diamond Light Source (Harwell, UK), respectively.

The diffraction data were indexed and integrated with DENZO, and merged with SCALEPACK.^34^ The statistics of the data are shown in Table 1. The crystals belong to the orthorhombic space group *P*2_1_2_1_2_1_, with one protein molecule per asymmetric unit and an approximate bulk solvent content of 44%. The structure of the PBP3/carbenicillin complex was solved by molecular replacement using the coordinates of *N. gonorrhoeae* PBP2 (PDB code 3equ)^4^ as search model. The starting model shares a 34% sequence identity with *P. aeruginosa* PBP3 and lacks the 72 residues that form the head subdomain. The orientation and position of the model in the PBP3 crystal were determined using the program MOLREP^35^ and refined with rigid-body refinement using data from 30 Å to 4.0 Å. The model was then subjected to restrained maximum-likelihood refinement with REFMAC using all data up to 2.3 Å resolution.^36^ The 2*F*_o_ − *F*_c_ map calculated at this stage showed well-defined electron density for the transpeptidase domain, which allowed the majority of residues within the domain to be built with confidence; the electron density for the N-terminal domain, especially for the missing head subdomain, was poor and fragmented, but gradually improved after each round of refinement and model rebuilding with Coot.^37^ Several rounds of refinement and model rebuilding led to a model of 498 residues (out of 543), with good stereochemistry and an *R*-factor of 0.196 (*R*_free _= 0.250) for all data up to 2.3 Å resolution. The structure of the PBP3/carbenicillin complex was then used to determine the structures of the apo-enzyme and the ceftazidime complex. The statistics for the final refined structures are given in Table 1.

### Accession numbers

The atomic coordinates and structure factors for apo-PBP3, the PBP3/carbenicillin complex, and the PBP3/ceftazidime complex have been deposited in the PDB under accession codes 3oc2, 3ocl, and 3ocn, respectively.

The following are the supplementary materials related to this article.

Fig. S1 Structures of carbenicillin [(2*S*,5*R*,6*R*)-6-{[carboxy(phenyl)acetyl]amino}-3,3-dimethyl-7-oxo-4-thia-1-azabicyclo[3.2.0] heptane-2-carboxylic acid] and ceftazidime [(6*R*,7*R*,*Z*)-7-(2-(2-aminothiazol-4-yl)-2-(2-carboxypropan-2-yloxyimino)acetamido)-8-oxo-3-(pyridinium-1-ylmethyl)-5-thia-1-aza bicyclo[4.2.0] oct-2-ene-2-carboxylate]. The asterisk marks the position where the catalytic serine attacks the drug.

## Figures and Tables

**Table 1:** X-ray data collection and refinement statistics

*Data collection*			
X-ray source	Diamond I04	ESRF ID23-2	Diamond I02
Data set	PBP3 native	PBP3/carbenicillin	PBP3/ceftazidime
Wavelength (Å)	1.0000	0.8726	0.9750
Space group	*P*2_1_2_1_2_1_	*P*2_1_2_1_2_1_	*P*2_1_2_1_2_1_
Unit cell parameters *a*, *b*, *c* (Å)	66.76, 81.51, 85.94	69.48, 83.44, 89.85	67.08, 81.81, 87.79
Resolution range (Å)	30.0–2.00 (2.07–2.00)	30.0–2.40 (2.49–2.40)	30.0–2.60 (2.69–2.60)
Unique reflections	32,803 (3150)	21,168 (2081)	15,047 (1331)
Completeness (%)	99.6 (96.7)	99.6 (99.9)	98.6 (89.3)
Redundancy	10.4 (5.7)	5.4 (4.9)	5.6 (3.7)
〈*I*/σ(*I*)〉	23.9 (3.0)	8.6 (1.9)	11.9 (2.5)
*R*_merge_	0.143 (0.575)	0.172 (0.582)	0.166 (0.545)

*Refinement statistics*			
Resolution range (Å)	30.0–2.00	30.0–2.40	30.0–2.60
Number of reflections (working/test)	31,100/1649	19,881/1090	14,178/764
*R*-factor^a^ (*R*_work_/*R*_free_)	0.171/0.213	0.195/0.242	0.207/0.267
Number of atoms (protein/other)	3787/220	3801/158	3841/69
RMSD on bond lengths (Å)	0.007	0.007	0.006
RMSD on bond angles (°)	1.1	1.1	1.0
Mean *B*-factor (protein/other) (Å^2^)	32/38	30/33	21/16
Ramachandran plot			
Residues in preferred regions (%)	469 (95.7)	474 (96.9)	373 (88.2)
Residues in allowed regions (%)	21 (4.3)	14 (2.9)	46 (10.9)
Residues in disallowed regions (%)	0 (0.0)	1 (0.2)	1 (0.2)

a *R*_work_ and *R*_free_ are defined by *R* = ∑_*hkl*_‖*F*_obs_| − |*F*_calc_‖/∑_*hkl*_|*F*_obs_|, where *hkl* are the indices of the reflections (used in refinement for *R*_work_; 5%, not used in refinement, for *R*_free_), and *F*_obs_ and *F*_calc_ are the structure factors deduced from measured intensities and calculated from the model, respectively.

**Table 2:** A comparison of class B PBP structures

PDB code	Number of C^α^ equivalences	Identical sequence	RMSD (Å)	Rotation (Å)	Translation (Å)
*Overlap of the C-domain onto PBP3 native (residues 222–558)*					
2wad-A	267	59	1.7	NA	
1rp5-A	293	75	1.9	NA	
1mwr-A	262	57	2.0	NA	
3equ-A	302	126	1.5	NA	

*Overlap of the N-terminal subdomain (residues 50–79 and 150–221)*					
2wad-A	84	20	1.8	24.9	1.1
1rp5-A	97	28	1.3	2.5	0.4
1mwr-A	71	24	1.4	17.0	1.0
3equ-A	90	41	1.7	8.9	0.1

*Overlap of the head subdomain (residues 80–149)*					
2wad-A	60	8	2.5	28.7	1.0
1rp5-A	67	7	1.9	60.3	2.1
1mwr-A	60	11	2.1	76.6	0.3
3equ-A					

NA, not applicable. The structures of the C-terminal transpeptidase domain, N-terminal β-stranded subdomain, and N-terminal β-stranded head subdomain were sequentially aligned to PBP3 using SHP.^23^ The results show the RMSD for C^α^ atoms, and the rotation and translation distances required to overlay all of the domains. In each case, molecule A in the PDB entries for the following structures was used for the comparison: *S. pneumoniae* PBP2b (PDB code 2wad), *S. pneumonia* PBP2x (PDB code 1rp5), *S. aureus* PBP2a (PDB code 1mwr), and *N. gonorrhoeae* PBP2 (PDB code 3equ).

## Footnotes

† ftp://ftp.sgc.ox.ac.uk/pub/biophysics
