# Supplementary material for: Crystal Structures of Penicillin-Binding Protein 3 from Pseudomonas aeruginosa: Comparison of Native and Antibiotic-Bound Forms
Source: J Mol Biol. 2011 Jan 7;405(1-3):173–84. doi: 10.1016/j.jmb.2010.10.024 (PMC3025346; doi:10.1016/j.jmb.2010.10.024)
Supplement: Fig. S1 — Structures of carbenicillin [(2S,5R,6R)-6-{[carboxy(phenyl)acetyl]amino}-3,3-dimethyl-7-oxo-4-thia-1-azabicyclo[3.2.0] heptane-2-carboxylic acid] and ceftazidime [(6R,7R,Z)-7-(2-(2-aminothiazol-4-yl)-2-(2-carboxypropan-2-yloxyimino)acetamido)-8-oxo-3-(pyridinium-1-ylmethyl)-5-thia-1-aza bicyclo[4.2.0] oct-2-ene-2-carboxylate]. The asterisk marks the position where the catalytic serine attacks the drug. [file mmc1.doc]

**Supplementary material.**

Figure S1. Structures of carbenicillin [(2*S*,5*R*,6*R*)-6-{[carboxy(phenyl)acetyl]amino}-3,3-dimethyl-7-oxo-4-thia-1-azabicyclo[3.2.0] heptane-2-carboxylic acid] and ceftazidime [(6*R*,7*R*,*Z*)-7-(2-(2-aminothiazol-4-yl)-2-(2-carboxypropan-2-yloxyimino)acetamido)-8-oxo-3-(pyridinium-1-ylmethyl)-5-thia-1-aza bicyclo[4.2.0] oct-2-ene-2-carboxylate]. The * marks the position where the catalytic serine attacks the drug.
